# Worth of a Wag

**Published:** 1873-08

**Authors:** 


### WORTH OF A WAG.

THE poor old Dutchman would not take five dollars for his ugly, worthless dog. He would willingly sell the dog for a dollar, but "I can't sell the vag of his tail ven I comes home at night!" When the poor fellow came home at the close of the day, wearied, dispirited and sad, the ugly little creature would always welcome him with such sincere demonstrations of joy and affection, the wag of the tail being the exponent of the same, that he was unwilling to part with the dog for any price that a man would give for it.

In every department of life persons are found in whom quite a number of good qualities or characteristics are absent, yet there is some virtue present which more than compensates for all the deficiencies. The wife of the President of the United States is "cross-eyed," compelling her to be all the time looking two ways for Sunday, and yet she is a woman of such sterling worth—of such unpretending deportment—so confiding, and so affectionate as a wife and mother, that the defect is no drawback in the estimation of either husband or child, and, very possibly, has a positive value in their minds.

You will often see a husband and wife together—the gentleman an almost perfect specimen of a man, the wife a poor, puny, sickly thing, more like a corpse than anything else—always dying but never dead—and yet he is observed to treat her with as much consideration, and tenderness, and affection as if she were a queen; on inquiry, it will be found that she has characteristics which have endeared her to him, to an extent which makes her, in his estimation, beyond price.

At other times an elegant woman—cultivated, refined and accomplished—is mated with a husband so plain in face, so common in dress, so unattractive in person, and so ungainly in gait and deportment, you involuntarily exclaim, "How can that woman live a single hour with such a man?" and yet, a nearer view of him will disclose characteristics which are worth more than gold.

You deal with a merchant; he is close, stingy, rough and unaccommodating—even repulsive sometimes—and yet you go to his store year after year in preference to any other, and why? His goods are always sold at a fair price, and are of a better quality than you can get anywhere else.

Your dressmaker talks you to death; she is insufferably tedious, and so particular that she will measure and fit and alter a dozen times, and, may be, often keeps you standing until you are "ready to drop;" and yet you have given her your custom for years and years, because her style is becoming to you, her fits are perfect, and her work is always well done.

Your chambermaid is many times very insulting; you can scarcely get a civil answer from her; and she has such supercilious ways, too! but you keep her one, five, ten years, because she is always so tidy; she is the best cleaner in the world, and you never have to tell her how or what should be done a second time. She is thorough in everything. And the cook. She is so cross—so slow—never seems to do anything willingly; if you enter her kitchen once in a month she never fails to "look daggers at you;" if you send an order that is unexpected or unusual, she is pretty sure to send you up word that she "won't do it, at all, at all." Your visitors never fail to give you a very prompt and decided statement, "I wouldn't keep her a minute." "Why don't you send her off?" That's the way they do, with the result that they are always changing—always have strange people about them, and very often have to cook themselves. But you have kept your cook "going on twenty years," because she is the "best bread maker in the world;" your roasts and your porter house steaks come on the table done to a turn, and "fit for a king." Dinner is always ready at the moment, but never by any possibility ten minutes before the time, even if the house was burning down; for she "won't be hurried;" she never goes anywhere but to church; she has no relations; never tells a lie; never wastes; never steals; but is as "honest as the day is long," and her kitchen is the perfection of neatness, cleanliness and order; the very floor seems as clean as the snow-white sanded table, and as for her washing, "she can't be beat." The practical conclusion is, that although everybody has not a tail, yet every one has his wag; and many lack the wisdom of the poor old Dutchman, whose little dog's wag was worth to him all the world besides.

